# Antioxidant and Hypolipidemic Activities of Acid-Depolymerised Exopolysaccharides by *Termitomyces albuminosus*

**DOI:** 10.1155/2019/8915272

**Published:** 2019-09-08

**Authors:** Huajie Zhao, Xiuxiu Wang, Xinchao Liu, Jianjun Zhang, Luzhang Wan, Le Jia

**Affiliations:** ^1^Institute of Agricultural Resources and Environment, Shandong Academy of Agricultural Science, Jinan 250100, China; ^2^College of Life Science, Shandong Agricultural University, Taian 271018, China

## Abstract

The acid-depolymerised exopolysaccharides (ADES) of *Termitomyces albuminosus* were obtained, and the major fraction of ADES1 was isolated and purified by DEAE-52 cellulose anion-exchange column chromatography. Physicochemical characterizations showed that ADES1 was an *α*- and a *β*-configuration with the molecular weight of 2.43 kDa, containing (1→3, 4)-linked-Glc*p*, (1→4)-linked-D-Glc*p*, (1→3)-linked-D-Xyl*p*, (1→4)-linked-D-Man*p*, T-Glc*p*, (1→6)-linked-D-Gal*p*, and (1→4)-linked-L-Ara*p*. The *in vivo* assays showed that ADES1 could reduce lipid levels in the serum and liver, decrease serum enzyme activities, and improve antioxidant enzyme activities and p-AMPK*α* expressions in hyperlipidemic mice, which were also confirmed by histopathological observations. These data indicated that ADES1 might be considered as a novel substance to treat and prevent hyperlipidemia and as a hepatoprotective agent.

## 1. Introduction

Hyperlipidemia is a complex status in which low-density lipoprotein cholesterol (LDL-C), total cholesterol (TC), and/or triglyceride (TG) levels are elevated, while the high-density lipoprotein cholesterol (HDL-C) level is lowered clinically [1, 2]. Accumulated reports have demonstrated that hyperlipidemia can be caused by receiving excess high-fat/high-cholesterol food [3]. Based on previous academic literatures, the oxygen free radicals, which were regarded as reactive oxygen species (ROS) and generated in the oxidative metabolic process incessantly, can accelerate the formations and progress of hyperlipidemia and its complications [4–7]. ROS in the body can be neutralized *via* endogenous mechanisms including enzymatic and nonenzymatic antioxidant systems, but the nonneutralized ROS will destroy the intracellular antioxidant system, thereby leading to protein denaturation, DNA damage, and lipid peroxidation [8]. Therefore, it is essential to supplement extra antioxidants for therapies against the free radical-induced lesions [9]. However, most of the currently available statins and fibric acid derivatives in clinical practice have been demonstrated to cause adverse effects for the prolonged treatment patients such as myopathy, rhabdomyolysis, renal insufficiency, liver enzyme elevations, and gastrointestinal side effects [10]. Hence, exploiting new natural drugs against hyperlipidemia is still significative. In recent years, mushroom polysaccharides from the fruiting body, fermentation liquor, and mycelia have been reported to be useful for lowing hyperlipidemia, such as *Pholiota nameko*, *Lachnum* YM281, and *Catathelasma ventricosum* polysaccharides owing to their higher antioxidant and preoxidant abilities [11–13].

*Termitomyces albuminosus*, a well-known wild mushroom and mostly distributed in the tropical areas of Africa and Asia, showed a symbiotic relationship with termites and are difficult to be artificially cultivated [14, 15]. The phytochemical assays indicated that *T. albuminosus*-containing polysaccharides, coumarin, saponins, cerebrosides, and ergosterol are usually considered to exert medicinal properties on increasing the thinking process, enhancing the stomach absorption functions, treating hemorrhoids, and guarding against intestinal carcinoma [16]. In the modern fermentation technology, the submerged fermentation of mushrooms, in particular for the symbiotic ones, showed superior and alternative applications comparing with the cultivations. Meanwhile, previous reports had shown that the polysaccharides from the mycelia and fermentation liquor of *T. albuminosus* possess antioxidant, antihyperlipidemic, hepatoprotective, analgesic, and anti-inflammatory activities [16–18]. And Liu et al., Gao et al., and Ren et al. have also reported that the polysaccharides after acidic hydrolysis possess stronger biological activities comparing with the normal ones [19–21]. Therefore, the present treatment on EPS was acidic hydrolysis. In this work, ADES1 was prepared and its antioxidant, hypolipidemic, and hepatoprotective activities in high-fat emulsion- (HFE-) induced hyperlipidemia mice were evaluated.

## 2. Materials and Methods

### 2.1. Chemicals

The strain of *T. albuminosus* used in this experiment was acquired from our laboratory. Kunming strain male mice were purchased from Taibang Biologic Products Co. Ltd. (Taian, China). The diagnostic kit assaying activities of superoxide dismutase (SOD), glutathione peroxidase (GSH-Px), and catalase (CAT), as well as the contents of malondialdehyde (MDA), total cholesterol (TC), and triglyceride (TG), were purchased from Nanjing Jiancheng Bioengineering Co. Ltd. (Nanjing, China). The standard monosaccharide samples were provided by Merck Company (Darmstadt, Germany). DEAE-52 cellulose was purchased from Sigma Chemicals Company (St. Louis, USA). All other chemicals used in the present work were analytical reagent grade and supplied by local chemical suppliers.

### 2.2. Culture

The liquid seed cultivation of *T. albuminosus* was processed in a 1 L filter flask containing 500 mL of 200 g/L potato, 20 g/L glucose, 1.5 g/L KH_2_PO_4_, and 1 g/L MgSO_4_·7H_2_O at 25°C. After one week, the liquid seed was inoculated into a fermentation tank (100 L, Xianmin, China) for 10 days.

### 2.3. Extraction of EPS

The fermentation broth of *T. albuminosus* was collected by filtration, concentrated, and precipitated with three volumes of ethanol (95%, *v*/*v*) for a night at 4°C. After centrifugation (3000 rpm, 10 min), the resulting precipitate was deproteinized by the Sevag method with chloroform/n-butanol (5 : 1, *v*/*v*) [22], dialyzed and lyophilized to offer EPS powder.

### 2.4. Preparation and Purification of ADES

The preparation of ADES was performed using the method reported by Ma et al. [23]. The EPS powder (0.5 g) and H_2_SO_4_ solution (1 mol/L, 10 mL) were mixed and incubated in boiling water for 8 h. The reaction mixture was centrifuged (3000 rpm, 10 min), neutralized with 2 mol/L NaOH solution, dialyzed, and lyophilized to obtain ADES powder.

The ADES solution (0.1 g/mL) was poured into a DEAE-cellulose column (1.6 × 20 cm) and eluted at a flow rate of 1.5 mL/min with distilled water and gradient NaCl solutions of 0.0, 0.1, 0.2, 0.3, and 0.5 mol/L, monitored by the phenol-sulfuric acid method [24]. The major fraction of ADES1 was collected for further analysis.

### 2.5. Preliminary Characterization Analysis of ADES1

The monosaccharide analysis was conducted using the methods reported by Luo et al. [25]. The processed product was loaded onto a capillary gas chromatography (GC) column of Rtx-1 (30 mm × 0.25 mm × 0.25 *μ*m) equipped with a flame ionization detector (FID), using L-rhamnose (L-Rha), D-ribose (D-Rib), L-arabinose (L-Ara), D-xylose (D-Xyl), D-mannose (D-Man), D-galactose (D-Gal), and D-glucose (D-Glc) as the internal standard.

The molecular weight (Mw) was determined using high-performance liquid chromatography (HPLC) by a previously reported method [26]. The sample (20 *μ*L, 2 mg/mL) was injected into an Atlantis C18 column (250 mm × 4.6 mm × 5 *μ*m) at a flow rate of 1 mL/min. The standard dextrans were used to draw the standard curve, and Mw was analyzed by Agilent GPC software.

The FT-IR was analyzed using a 6700 Nicolet Fourier-transform infrared spectrophotometer (Thermo Fisher Scientific Co., USA) in the frequency range of 4000–500 cm^−1^ after mixing the ADES1 with a potassium bromide disc.

The methylation analysis of ADES1 was carried out using a precise method [27]. The mixture including 10 mg ADES1, NaOH (1 g), and 10 mL anhydrous dimethyl sulfoxide was conducted by an ultrasonic wave for 30 min. 3 mL methyl iodide was added to the above reaction mixture, incubated for 1 h at 25°C, kept in darkness at room temperature for 8 h, and finally terminated using 3 mL distilled water. The reaction product was extracted with trichloromethane and dried on a rotary evaporator to obtain methylated ADES1, which was then hydrolyzed, reduced, and acetylated. The produced partially methylated alditol acetate was analyzed by gas chromatography-mass spectrometry (GC-MS).

The ADES1 was dissolved in deuterated water. ^1^H and ^13^C nuclear magnetic resonance (NMR) was recorded by *via* a Bruker AV-300 spectrometer operating at 25°C.

### 2.6. Hypolipidemic Effect Analysis

#### 2.6.1. High-Fat Emulsion Preparation

The oil phase (25 g lard oil, 10 g cholesterol, 1 g methylthiouracil, and 25 mL Tween-80) and the water phase (30 mL distilled water, 20 mL propylene glycol, and 2 g sodium deoxycholate) were mixed to give the high-fat emulsion.

#### 2.6.2. Animal Experiment

All experiments were performed according to the rules considering animal experiments and the accepted ethical principles of the Shandong Agricultural University Committee. The sixty male Kunming mice (20 ± 2 g) were housed in the animal room under standardized conditions (temperature 22 ± 1°C, relative humidity 50 ± 5%, and a 12 h light/dark cycle) and given free access to food and water *ad libitum*.

After a 7-day acclimatization period, all mice were randomly divided into six groups (10 mice in each group) including one normal saline (NS) group receiving normal saline, one high-fat emulsion (HFE) group receiving alternating daily gavages of normal saline and high-fat emulsion, one simvastatin (SI) group receiving 200 mg/kg simvastatin, and three polysaccharide-treated groups composed of 100, 200, and 400 mg/kg groups receiving alternating daily gavages of the high-fat emulsion and polysaccharides.

All mice were fasted for one night, weighed, and euthanized at the fortieth day. The blood samples were separated from the retrobulbar vein, and the serum was collected by centrifugation at 3000 rpm for 15 min. The obtained serum was used for the evaluations of aspartate aminotransferase (AST) and alanine aminotransferase (ALT) activities and LDL-C, TC, TG, and HDL-C levels. Livers were removed immediately after euthanasia, weighed, homogenized (1 : 9, *w*/*v*) in phosphate buffer (0.2 mol/L, pH 7.4, 4°C), and centrifuged at 3000 rpm for 10 min, and then liver homogenates were used for assaying hepatic TC, TG, and MDA levels and SOD, CAT, and GSH-Px activities. Furthermore, the liver slices, which were prepared by soaking in 4% formalin, embedding in paraffin, cutting into slices, and staining with hematoxylin and eosin sequentially, were examined using a microscope of ×400 magnifications.

#### 2.6.3. Western Blotting Analysis

The liver tissue of mice (0.1 g) was added to the mixture including RIPA lysis buffer (1 mL) and phosphatase inhibitor cocktail (10 *μ*L), homogenized by a glass homogenizer (2 mL), incubated for 20 min in ice water, and centrifuged at 14000 rpm for 10 min to obtain the supernatant, which was mixed with loading buffer (5x) and boiled for 10 min. The obtained protein solution was separated using 10% sodium dodecyl sulfate polyacrylamide gel electrophoresis. The separated proteins were electrophoretically transferred to polyvinylidene difluoride membranes (0.45 *μ*m), which were blocked with 5% (*w*/*v*) BSA in TBST at room temperature for 2 h. The enclosed membrane was incubated with primary rabbit antibodies (anti-p-AMPK*α*, 1 : 1000; anti-GAPDH, 1 : 1000, Cell Signaling Technology Inc., Boston, USA) overnight at 4°C and then employed with peroxidase-conjugated goat anti-rabbit as secondary antibodies (1 : 5000) (Absin Bioscience Inc., Shanghai, China) at room temperature for 1 h. Protein bands were visualized using the ECL Western Blotting System (GE Healthcare Life Sciences) and band density quantified using an AlphaImager imaging system (Alpha Innotech Corporation).

### 2.7. Acute Toxicity Evaluation

The acute toxicity evaluation was evaluated using a previously reported method [28]. Briefly, twenty mice were randomized into two groups (ten mice in each group) including the normal control (NC) group and the toxicity evaluation group. The NC group was treated with NaCl (0.9%) solution, and the toxicity evaluation group was administrated with increasing dosages of 500, 1000, 1500, and 2000 mg/kg samples. During the whole evaluation, all mice were fed a normal chow diet with continuous observation for gross behavioral changes, toxic symptoms, and mortality for 48 h.

### 2.8. Statistical Analysis

SPSS software was used for statistical analyses, and all data were expressed as means ± SD (standard deviations). Differences among experimental groups were considered as statistically significant if *P* < 0.05 by one-way ANOVA of Duncan's multiple range tests.

## 3. Results and Discussion

### 3.1. Purification and Structural Characterization

**Figure 1:**
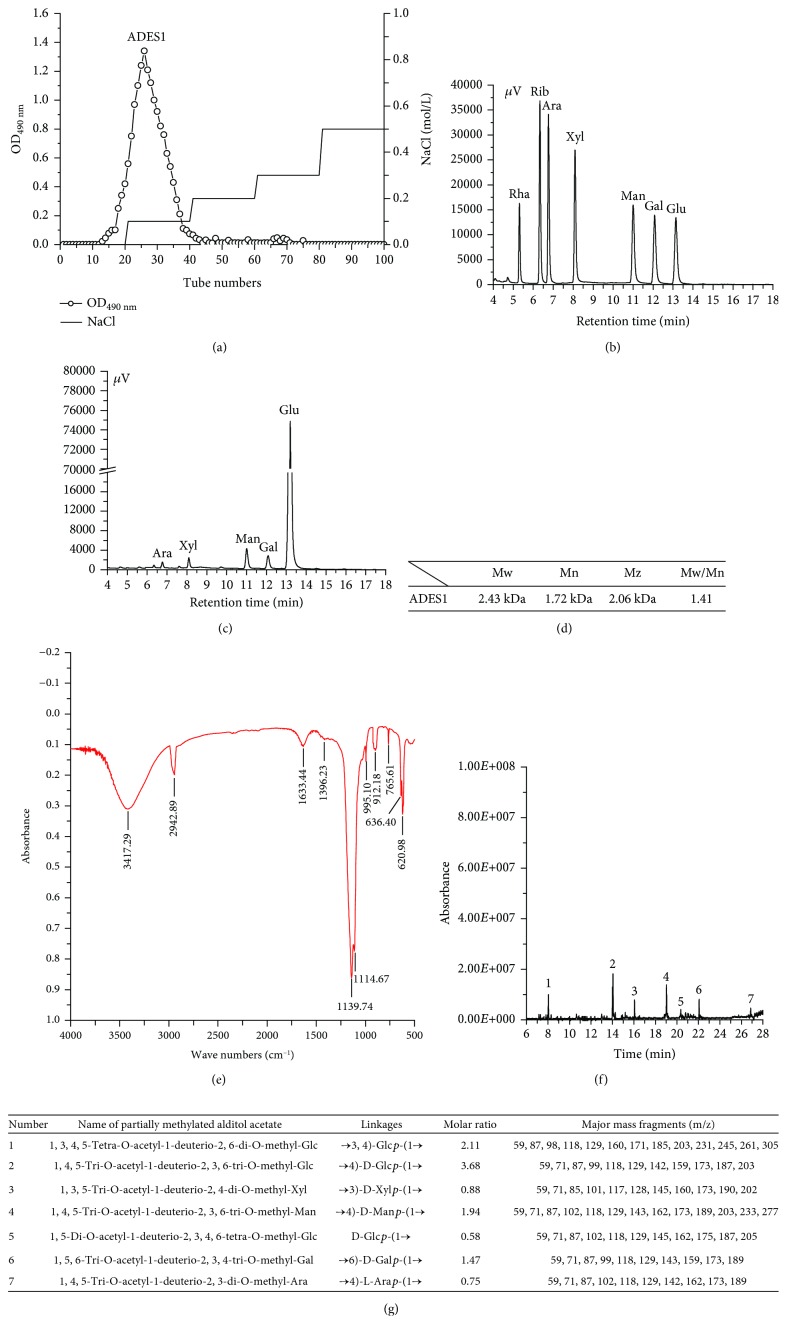
Purification and physicochemical analysis of ADES1: DEAE-52 cellulose chromatogram (a); GC analysis (b, c); molecular weights (d); FT-IR analysis (e); GC-MS (f, g).

The single peak was found in Figure 1(a) by DEAE-52 chromatography, manifesting that ADES had only one major fraction. This fraction was collected and named as ADES1, which was used for further studies.

Based on the monosaccharide composition analysis (Figures 1(b) and 1(c)), ADES1 was composed of L-Ara, D-Xyl, D-Man, D-Gal, and D-Glc with a molar ratio of 1.00 : 1.10 : 2.22 : 4.16 : 16.01, showing that ADES1 was a heteropolysaccharide and Glc was the main sugar unit. However, monosaccharide compositions of ADES1 were different neither from those of mycelia polysaccharides and EPS from *T. albuminosus* [17, 18] nor from *Flammulina velutipes*, *Grifola frondosa*, and *Inonotus obliquus* [23, 29, 30]. The differences might be related to the methods of extraction and processing, origin, strains, culture medium, and so on.

Based on the results of the HPLC analysis, the Mw, number-average molecular weight (Mn), and *z*-average molecular weight (Mz) of ADES1 were 2.43 kDa, 1.72 kDa, and 2.06 kDa, respectively (Figure 1(d)).

The FT-IR spectrum analysis showed the major functional groups and the chemical bonds of ADES1 (Figure 1(e)). A wide and strong peak at 3417.29 cm^−1^ was caused by the –OH stretching vibrations, and the absorption peak at around 2942.89 cm^−1^ was attributed to the C–H stretching and bending vibrations [31]. Two bands at 1633.44 cm^−1^ and 1396.23 cm^−1^ were attributed to asymmetrical and symmetrical stretching vibration of the carboxylate anion group (C=O); the absorption band 1633.44 cm^−1^ was also assigned to the bending vibration of water [32–34]. The absorption peaks at 1139.74 cm^−1^ and 1114.67 cm^−1^ during the range from 1200 cm^−1^ to 1000 cm^−1^ manifested the presence of a pyranose [35]. The characteristic bends at 912.18 cm^−1^ and 765.61 cm^−1^ resulted from the stretching vibrations of *β*-D-pyranoid glucose and *α*-isomers of pyranose, respectively [36]. These data showed ADES1 was a polysaccharide with *α*- and *β*-configurations.

The total ion chromatogram and linkage pattern of ADES1 were summarized in Figures 1(f) and 1(g). According to the retention time and comparison with the Complex Carbohydrate Research Center database and relevant references, the results showed that ADES1 was composed of seven major glycosidic linkages including (1→3, 4)-linked-Glc*p*, (1→4)-linked-D-Glc*p*, (1→3)-linked-D-Xyl*p*, (1→4)-linked-D-Man*p*, nonreducing terminal of Glc*p*, (1→6)-linked-D-Gal*p*, and (1→4)-linked-L-Ara*p* in a molar ratio of 2.11 : 3.68 : 0.88 : 1.94 : 0.58 : 1.47 : 0.75. These results showed a good correlation between terminal and branched residues.

**Figure 2:**
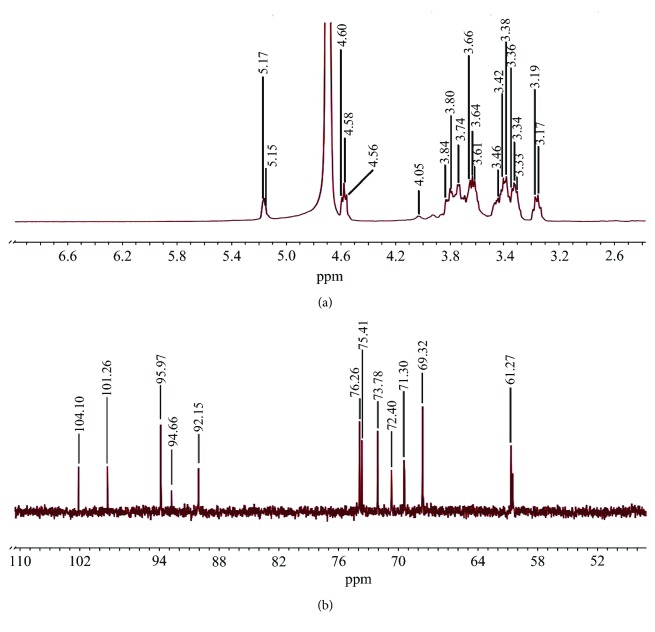
NMR analysis: ^1^H NMR (a); ^13^C NMR (b).

The ^1^H NMR spectrum of a typical polysaccharide was mainly congested during a confined region of 3-5 ppm [37]. As displayed in Figure 2(a), the ^1^H NMR spectrum of ADES1 revealed five signals of anomeric protons at 5.17, 5.15, 4.60, 4.58, and 4.56 ppm, manifesting that ADES1 was made up of five monosaccharides and was both an *α*- and a *β*-configuration polysaccharide [36, 38], which was verified by the above results of GC analysis and FT-IR analysis, respectively. Furthermore, the signals (4.5–5.2 ppm) were allocated to the anomeric region of H–1 and numerous signals (3.17–4.05 ppm) were ascribed to atoms H2–H6.

The ^13^C NMR spectrum peaks of ADES1 principally were crowded during a narrow region from 60 ppm to 110 ppm, which was the typical allocation of the polysaccharides' NMR signals (Figure 2(b)) [39]. The five signals at 92.15, 94.66, 95.97, 101.26, and 104.1 ppm during the range from 90 to 110 ppm showed that ADES1 consisted of five monosaccharides and was an *α*- and a *β*-configuration polysaccharide [36, 40]. In addition, the signals (92.15–104.10 ppm) were assigned to the anomeric region of H–1, and numerous signals (61.27–76.26 ppm) were attributed to atoms C2–C6.

Furthermore, Liu et al. had reported that the antioxidant and biological activities of polysaccharides were related to its characterizations [36]. Gao et al. had showed that Glc might play an important role in maintaining the antioxidant status [37]. Meantime, Chen et al. have showed that low-Mw polysaccharides possess superior biological activity, compared with high-Mw polysaccharides [34]. Meng et al. and Song et al. had reported that polysaccharides with *α*- and *β*-configurations possessed strong antioxidant, immunostimulatory, and hepatoprotective activities [29, 41]. These conclusions supported that ADES1 containing Glc with *α*- and *β*-configurations had antioxidant and other biological activities.

### 3.2. Hypolipidemic and Hepatoprotective Effects

#### 3.2.1. Effects of ADES1 on Body Weights

Differences of the body weights and liver indexes among all mice could be seen in Table 1. The body weight differences were not observed at the beginning experiment, and the final body weight of the HFE group was remarkably elevated than that of the NS group (*P* < 0.05), indicating that HFE could obviously induce mice obesity. Compared to the HFE group, the body weight increases of mice treated with ADES1 and SI were significantly suppressed (with all *P* < 0.05). Furthermore, the abnormal liver index induced by HFE was also improved by the administration of ADES1. These results demonstrated that ADES1 possessed a potential role in decreasing the HFE-induced body weight and liver index.

#### 3.2.2. Effects of ADES1 on Serum Lipids

**Figure 3:**
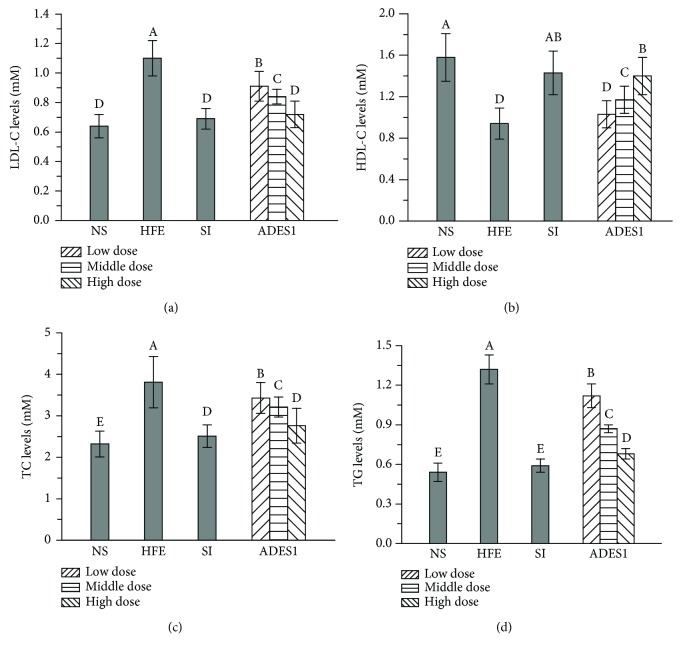
Effects of ADES1 on serum lipid profiles: (a) LDL-C levels, (b) HDL-C levels, (c) TC levels, and (d) TG levels. The values are reported as means ± SD. Bars with different letters are significantly different (*P* < 0.05).

To evaluate preventive effects of ADES1 on HFE-induced hyperlipidemia, the LDL-C, TC, TG, and HDL-C levels in serum were measured. Several literatures have shown that the existence of superfluous serum LDL (a main carrier of cholesterol transport) could be transferred to the endarterium and oxidized into oxidized LDL, which could reduce the specific membrane receptors' interaction with LDL and raise its duration in the bloodstream [4]. Therefore, the complex of LDL and cholesterol (LDL-C) which abnormally accumulated in the blood vessel walls resulted in the formation of an atherosclerotic plaque lesion [42]. Besides, high levels of serum TC and TG can aggrandize the blood viscosity and the risk of hyperlipidemia and atherosclerosis formation. On the contrary, HDL, an extraordinarily complex, dynamic, and heterogeneous granule demarcated by the density ranges from 1.21 g/mL to 1063 g/mL, is supposed to be “helpful” for lipid metabolism due to that it can take along the cholesterol/cholesterol ester from peripheral tissues/cells to the liver for catabolism *via* the reverse cholesterol transport pathway during blood circulation [17, 43]. And low levels of serum HDL-C can show the status of the most common lipid abnormality. Hence, regulating lipid metabolism disorder is deemed to be an effective method to retard/prevent the development of hyperlipidemia. As shown in Figure 3, an evident increase in the hepatic levels of LDL-C, TC, and TG and a remarkable decrease in the HDL-C levels of the HFE group were observed in comparison with those of the NS group (with all *P* < 0.05), making it clear that mice in the HFE group were exposed to an early lipid metabolism disorder. Unsurprisingly, the supplementations with ADES1 apparently mitigated the LDL-C, TC, and TG levels and significantly elevated the HDL-C level when compared to the HFE group. The LDL-C, TC, and TG levels in the H-ADES1 group reached 0.72 ± 0.07, 2.76 ± 0.42, and 0.68 ± 0.04 mM, which were 34.55%, 27.56%, and 48.48% lower than that in the HFE group, respectively, while the HDL-C level was 1.4 ± 0.18 mM, which was 48.94% higher than that in the HFE group. These results indicated that ADES1 had a stronger lipid-lowering ability.

#### 3.2.3. Effects of ADES1 on Hepatic Lipids

**Figure 4:**
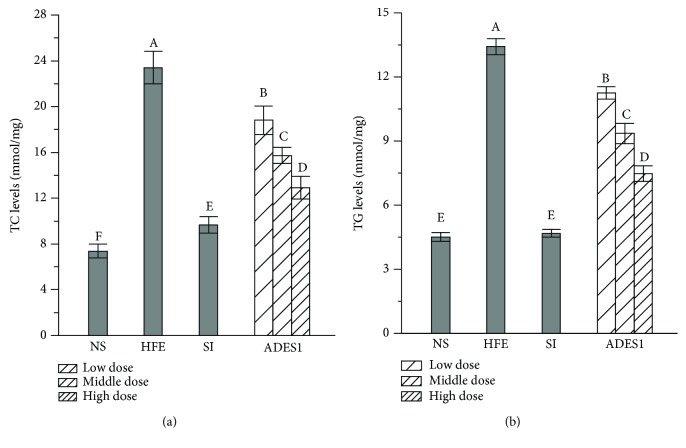
Effects of ADES1 on liver lipid profiles: (a) TC and (b) TG. The values are reported as means ± SD. Bars with different letters are significantly different (*P* < 0.05).

Some researchers have reported that the administration of HFE can obviously lead to the dysregulation of hepatic lipid metabolism characterized by high levels of TC and TG, which could reflect the fat gathered and the presence of lipochondrions on the surface of the hepatocyte, thereby attenuating liver function [11]. As displayed in Figures 3(b) and 4(a), hepatic lipid analysis displayed that TC and TG levels of mice in the HFE group were obviously elevated by 217.21% and 197.56%, respectively, compared to those in the NS group, declaring that HFE had successfully induced the abnormal metabolism of liver fat. Interestingly, the administration of ADES1 suppressed the elevation of TC and TG levels compared to the HFE group, demonstrating that ADES1 might be helpful to repair HFE-induced hepatic lipid metabolism disorders.

#### 3.2.4. Effects of ADES1 on p-AMPK*α* Expressions

**Figure 5:**
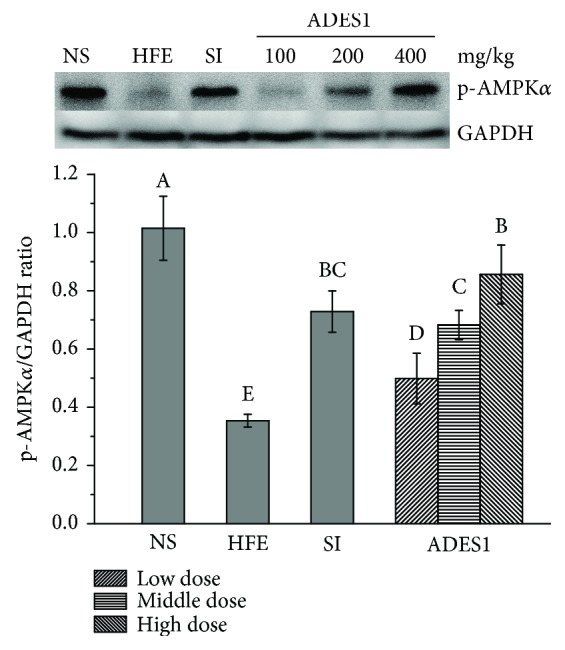
Effects of ADES1 on p-AMPK*α* expressions. The values are reported as means ± SD. Bars with different letters are significantly different (*P* < 0.05).

AMPK, a major regulator of lipid metabolism and an intracellular energy sensor, has been implicated in lipid and glucose homeostasis [44]. Activation of hepatic AMPK can increase the oxidation of fatty acid and simultaneously inhibit the synthesis of hepatic levels of fatty acid, TG, and TC [45]. In our work, the western blotting data showed that ADES1 elevated the level of the p-AMPK*α* expression, especially ADES1 at a dose of 400 mg/kg (Figure 5), indicating that ADES1 can activate AMPK to regulate the hepatic lipid metabolism system.

#### 3.2.5. Effects of ADES1 on the Antioxidant Status

Oxidative stress as an important contributor of the various pathological statuses such as cardiovascular, atherosclerosis, inflammation, cancer, drug toxicity, reperfusion injury, and neurodegenerative and liver diseases that could be induced by HFE could accelerate the radical formation that can damage the intrinsic antioxidant defense [18, 46]. Therefore, scavenging ROS is essential for reducing the level of oxidative stress. Antioxidant enzymes (SOD, GSH-Px, and CAT) could transform reactive oxygen molecules into nontoxic substances, thus building the first line of defense against ROS when oxidative stress occurs in the organism, and its possible mechanism is that SOD can convert superoxide radicals into hydrogen peroxide, which could then be broken down into H_2_O and O_2_ by GSH-Px and CAT, preventing the formation of ROS [47, 48].

**Figure 6:**
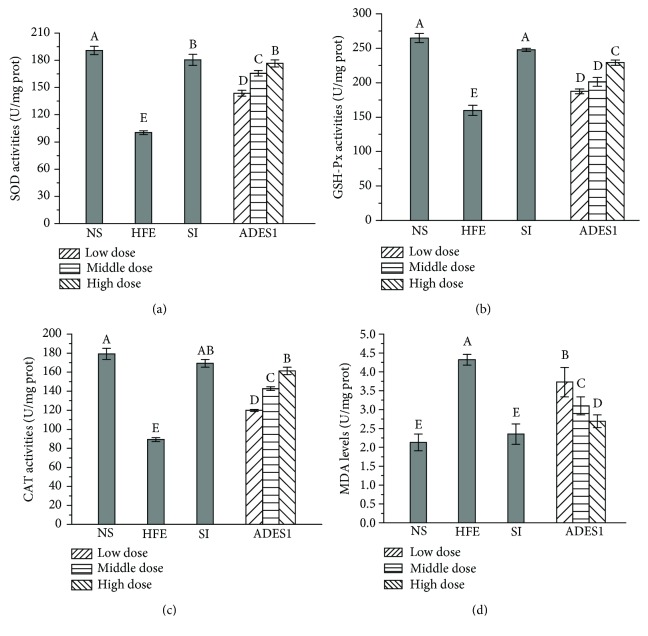
Effects of ADES1 on antioxidant status: (a) SOD activities, (b) GSH-Px activities, (c) CAT activities, and (d) MDA levels. The values are reported as means ± SD. Bars with different letters are significantly different (*P* < 0.05).

SOD is an important antioxidant enzyme that can respond to oxygen radicals and afford the greatest reaction to scavenge the superoxide anion radicals, thereby guarding against cellular damage [49]. Compared with that in the NS group (190.96 ± 4.46 U/mg prot), the SOD activity was significantly raised (*P* < 0.05, Figure 6(a)). The activities of SOD were 176.61 ± 3.95, 165.77 ± 3.01, and 143.66 ± 3.26 U/mg prot, which were 76.13%, 65.32%, and 43.27% higher than those of the HFE group at 400, 200, and 100 mg/kg per group, respectively.

GSH was readily oxidized to glutathione disulfide upon reaction with xenobiotic compounds, which was accompanied by decreasing hydrogen peroxide concentration, hydroperoxide, and xenobiotic toxicity, thereby preventing oxidative stress-induced cell damage [50]. The data showed that the GSH-Px activity of mice in the HFE group was lower than that in the NS group (*P* < 0.05, Figure 6(b)). Interestingly, the changes of GSH-Px activities which were increased by 43.50%, 25.78%, and 17.35% in the high-, middle-, and low-dose groups in comparison with those in the HFE group were dramatically mitigated with ADES1 intake.

As the most important enzyme which provides homeostasis for hydrogen peroxide, the physiological variation of CAT concentration in diverse tissues and organs gives rise to different steady-state levels of hydrogen peroxide concentration for the same rate of hydrogen peroxide generation [51]. As shown in Figure 6(c), the CAT activity of the HFE group was observably higher than that in the NS group (*P* < 0.05). The CAT activities treated with ADES1 at the dose of 100, 200, and 400 mg/kg were 119.85 ± 1.08, 142.61 ± 1.92, and 161.32 ± 3.85 U/mg prot, respectively.

As a secondary product of lipid peroxidation, the excess MDA can impede lipid metabolism and promote lipid peroxidation, which caused the oxidative stress occurring in cells [52]. Hence, it is critical to regulate lipid peroxidation to preserve the antioxidant defense system. The result showed that there was a distinct increase of the MDA level in the HFE group as compared to that in the NS group (Figure 6(d), *P* < 0.05), while the administration of ADES1 obviously reduced the MDA level (*P* < 0.05) as compared to that in the HFE group.

These results manifested that the ADES1 could enhance the activities of SOD, GSH-Px, and CAT and reduce the level of MDA in HFE-induced hyperlipidemia mice, especially ADES1 at the dose of 400 mg/kg, showing that ADES1 showed the potential ability against HFE-induced oxidative stress.

#### 3.2.6. Effects of ADES1 on Serum Enzymes

**Figure 7:**
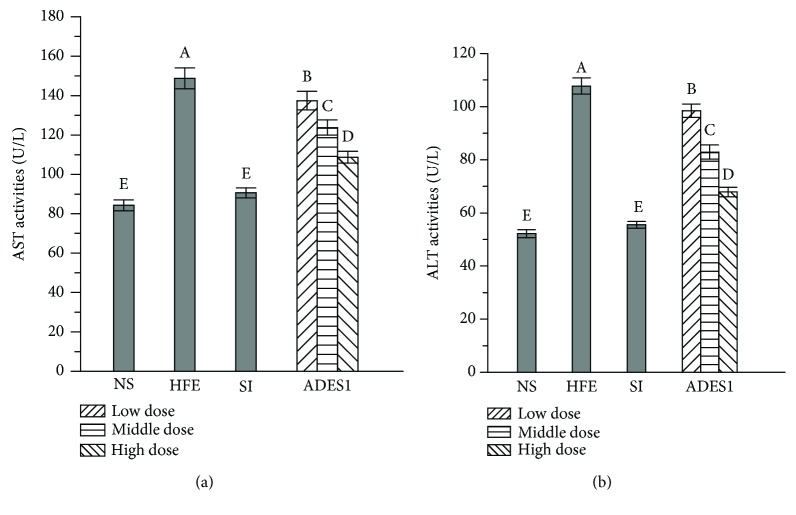
Effects of ADES1 on the serum enzymes: (a) AST and (b) ALT. The values are reported as means ± SD. Bars with different letters are significantly different (*P* < 0.05).

To assay the preferable effects of ADES1 on HFE-induced status changes of the liver tissue, the AST and ALT activities in serum were investigated (Figure 7). The AST and ALT, the enzymes found mainly in the hepatocytes, are the beneficial indicators to monitor the hepatic injury. Hence, the detected higher levels of these enzymes in the blood manifested that the liver is damaged, which is because the damaged hepatic cell membrane can cause AST and ALT in hepatic cells to leak into the blood circulation [12, 53]. The values of AST and ALT activities reached 148.73 ± 5.32 U/L and 107.76 ± 3.07 U/L in HFE group, respectively, and 84.26 ± 2.78 U/L and 52.17 ± 1.47 U/L in the NS group, respectively, indicating that HFE had caused liver injury. However, the administration of ADES1 dose-dependently significantly decreased the AST and ALT activities. The activities of AST and ALT in the high-dose group were 108.70 ± 2.92 U/L and 67.85 ± 1.79 U/L, respectively, which were 26.91% and 37.04% lower than the values observed in the HFE group, respectively. These results testified that ADES1 can ameliorate liver injury induced by HFE intake.

#### 3.2.7. Histopathological Observations

**Figure 8:**
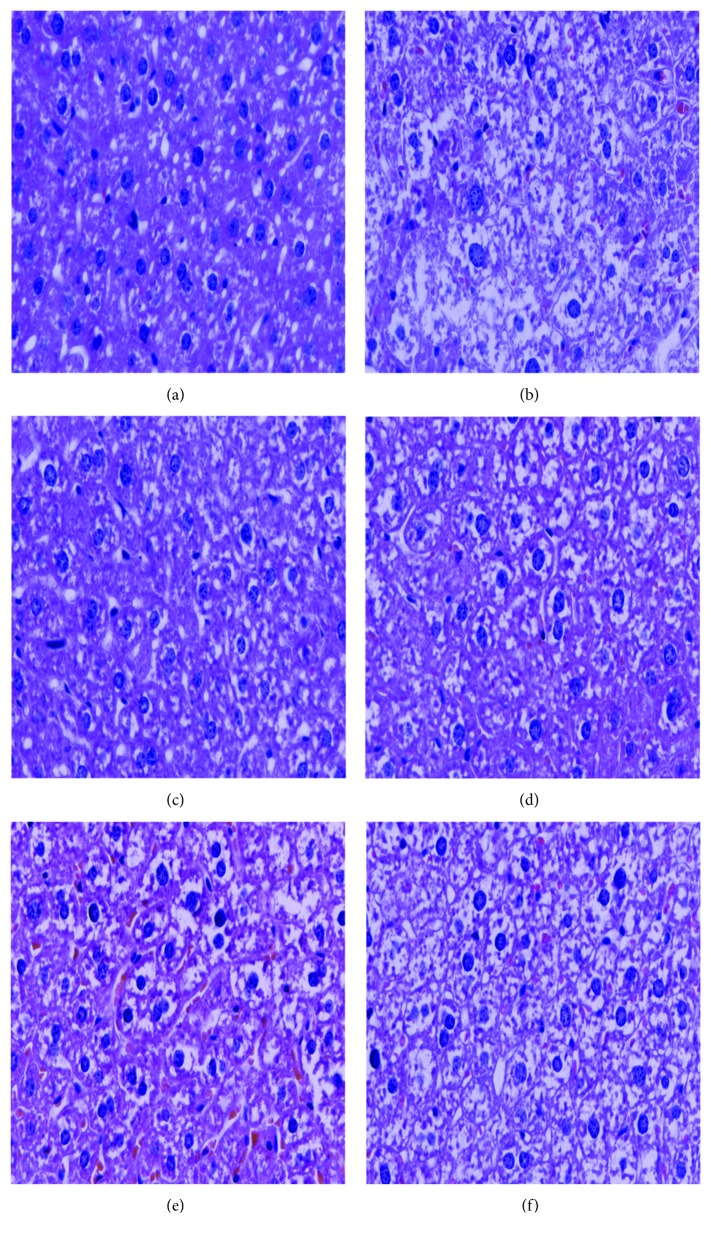
Optical micrographs of mouse liver sections (400x magnification). Liver sections from mice in the (a) NS, (b) HFE, (c) SI, (d) H-ADES1, (e) M-ADES1, and (f) L-ADES1.

The HFE-induced hepatic injury was exhibited by liver pathological changes characterized by variations of cellular morphology, hepatocyte arrangement, and fat. In the NC group (Figure 8(a)), complete hepatic cell morphology with well-preserved cytoplasm, nucleus, and nucleolus; orderly arranged hepatic cell cords; and no symptoms of fat degeneration were observed. In contrast, extreme swelling, large fat vacuoles, increased cell volume, mussy hepatic cell permutation, hepatic steatosis, and vesicular degeneration were observed in the HFE group (Figure 8(b)). The simvastatin treatment had positive effects on pathological injuries induced by HFE (Figure 8(c)). Interestingly, the abnormal histopathological changes were improved distinctly by the intervention with ADES1, especially H-ADES1, compared to the HFE group (Figure 8(d)–8(f)), making clear that ADES1 could weaken or treat the HFE-induced liver injury.

### 3.3. Acute Toxicity Evaluation

The apparent variations of behavior, autorhythmicity, and symptom of poisoning as well as deaths until the whole experiment ended in the toxicity evaluation group were not observed, indicating that ADES1 had no subacute toxicity on normal mice after high-dose intake.

## 4. Conclusions

ADES1 exposed *in vivo* antioxidant, hepatoprotective, and hypolipidemic activities in HFE-induced hyperlipidemic mice. The characterization analysis indicated that ADES1 was a typical polysaccharide with *α*- and *β*-configurations of L-Ara, D-Xyl, D-Man, D-Gal, and D-Glc with a molar ratio of 1.00 : 1.10 : 2.22 : 4.16 : 16.01. The studies on the structure-activity relationships of polysaccharides from *T. albuminosus* may provide understanding in hepatoprotective and hypolipidemic activities and may ultimately lead to the development of novel antihyperlipidemic agents.

## Figures and Tables

**Table 1:** Effects of ADES1 on body weights and liver indexes.

Group	Body weight (g)		Liver index (%)
	Initial	Final	
NS	20.12 ± 0.32	32.43 ± 0.23^D^	5.19 + 0.24^E^
HFE	19.79 ± 0.23	42.16 ± 0.91^A^	8.76 + 0.41^A^
SI	19.97 ± 0.38	36.82 ± 0.19^C^	6.01 + 0.32^D^
L-ADES1	20.08 ± 0.45	39.10 ± 0.17^B^	8.09 + 0.43^B^
M-ADES1	19.99 ± 0.29	37.18 ± 0.29^C^	7.12 + 0.28^C^
H-ADES1	20.00 ± 0.41	36.72 ± 0.30^C^	6.26 + 0.39^D^

Note: the values are reported as the means ± SD. Values with different superscript letters are significantly different (*P* < 0.05).

## Data Availability

The datasets used and/or analyzed during the current study will be available on reasonable request.

## Abbreviations

ADES: Acid-depolymerised exopolysaccharides
ALT: Alanine aminotransferase
L-Ara: L-Arabinose
AST: Aspartate aminotransferase
CAT: Catalase
EPS: Exopolysaccharide
FID: Flame ionization detector
FT-IR: Fourier-transform infrared
D-Gal: D-Galactose
GC: Gas chromatography
GC-MS: Gas chromatography-mass spectrometry
D-Glc: D-Glucose
GSH-Px: GSH peroxide
HDL-C: High-density lipoprotein cholesterol
HFE: High-fat emulsion
HPLC: High-performance liquid chromatography
LDL-C: Low-density lipoprotein cholesterol
D-Man: D-Mannose
MDA: Malondialdehyde
NC: Normal control
NS: Normal saline
NMR: Nuclear magnetic resonance
prot: Protein
L-Rha: L-Rhamnose
D-Rib: D-Ribose
ROS: Reactive oxygen species
SD: Standard deviations
SI: Simvastatin
SOD: Superoxide dismutase
TC: Total cholesterol
TG: Triglyceride
TE: Toxicity evaluation
D-Xyl: D-Xylose.
